# From cassava to gari: mapping of quality characteristics and end‐user preferences in Cameroon and Nigeria

**DOI:** 10.1111/ijfs.14790

**Published:** 2020-10-06

**Authors:** Robert Ndjouenkeu, Franklin Ngoualem Kegah, Béla Teeken, Benjamin Okoye, Tessy Madu, Olamide Deborah Olaosebikan, Ugo Chijioke, Abolore Bello, Adebowale Oluwaseun Osunbade, Durodola Owoade, Noel Hubert Takam‐Tchuente, Esther Biaton Njeufa, Isabelle Linda Nguiadem Chomdom, Lora Forsythe, Busie Maziya‐Dixon, Geneviève Fliedel

**Affiliations:** ^1^ Department of Food Science and Nutrition ENSAI University of Ngaoundéré Ngaoundere PO Box 455 Cameroun; ^2^ International Institute of Tropical Agriculture (IITA) Oyo Road Ibadan Nigeria; ^3^ National Root Crops Research Institute (NRCRI) Km 8 Umuahia‐Ikot Ekpene Road Umudike Abia State P.M.B. 7006 Nigeria; ^4^ International Institute of Tropical Agriculture (IITA) Eco‐regional Center HFS IRAD Main Road, Nkolbisson Yaoundé BP 2008 (Messa) Cameroon; ^5^ Natural Resources Institute University of Greenwich Central Avenue Chatham Maritime Kent ME4 4TB UK; ^6^ CIRAD UMR Qualisud Montpellier F‐34398 France; ^7^ Qualisud Univ Montpellier CIRAD, Montpellier SupAgro Univ d'Avignon Univ de La Réunion Montpellier F‐34398 France

**Keywords:** Cameroon, cassava, *eba*, *gari*, Nigeria, quality characteristics, root, user preferences, varieties

## Abstract

User’s preferences of cassava and cassava products along the value chain are supported by specific root quality characteristics that can be linked to root traits. Therefore, providing an evidence base of user preferred characteristics along the value chain can help in the functional choice of cassava varieties. In this respect, the present paper presents the results from focus group discussions and individual interviews on user preferred quality characteristics of raw cassava roots and the derived product, *gari*, – one of the major cassava products in Sub‐Saharan Africa – in major production and consumption areas of Cameroon and Nigeria. Choice of cassava varieties for farming is mainly determined by the multiple end uses of the roots, their agricultural yield and the processing determinants of roots that support their major high‐quality characteristics: size, density, low water content, maturity, colour and safety. Processing of cassava roots into *gari* goes through different technological variants leading to a *gari* whose high‐quality characteristics are dryness, colour, shiny/attractive appearance, uniform granules and taste. *Eba*, the major consumption form of gari in Cameroon and Nigeria, is mainly characterised by its textural properties: smoothness, firmness, stickiness, elasticity and mouldability. Recommendations are made, suggesting that breeding will have to start evaluating cassava clones for brightness/shininess, as well as textural properties such as mouldability and elasticity of cassava food products, for the purpose of supporting decision‐making by breeders and the development of high‐throughput selection methods of cassava varieties. Women are identified as important beneficiaries of such initiatives giving their disadvantaged position and their prominent role in cassava processing and marketing of gari.

## Graphical abstract

High quality characteristics of cassava roots, gari and eba (coloured boxes indicate high quality characteristics for each product)

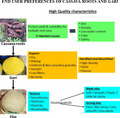

## Introduction

Cassava (*Manihot esculenta* Crantz), originating from South America, is one of the world’s main root crops and constitutes the most important staple of rural and urban households in Sub‐Saharan Africa (Spencer & Ezedinma, 2017; Petsakos *et al*., 2019). The tuberous root and its products feed more than 500 million African households with an average annual consumption of 100 kg of roots per person, with Nigeria being the world’s leading producer and consumer, with an annual production of 59.47 million metric tons, 65% of which is consumed locally (FAO, 2018a). In Cameroon, although cassava production (≈5 million tons) is ten times less than that of Nigeria, the root is the major crop in this country, with an increasing production yield (≈13–14 tons per ha in 2012–2013), comparable to values registered in Nigeria, both in terms of level of consumption and calory contribution of the roots (FAO, 1991; Spencer & Ezedinma, 2017). The importance of cassava as a staple food results mainly from the simplicity of its cultivation; its ability to grow on marginal land that are difficult to use for other crops; and its drought tolerance, which justifies its geographical expansion from its natural forest areas to the Sahelian zones (Nweke, 2004; IFAD, 2008; Funke *et al*., 2012; Ukwuru & Egbonu, 2013; Olanrewaju, 2016). In addition, at the level of subsistence agriculture, the root can be left in the ground and harvested piece meal, thus allowing a spread management of its food use. These advantages have confirmed cassava as a very important crop which is fast replacing traditional crops in some areas, gaining ground increasingly as an insurance crop against hunger and climate change and consequently constitutes an essential component of food security for African populations. Cassava is also a major cash crop for a large number of households and is sold fresh or after processing to generate income, often used by women for household purchases, children’s education, health and investment in business (Forsythe *et al*., 2016).

Cassava has two main forms of consumption in Africa: the peeled and cooked root absorbs about 30% of production and the remaining 70% is processed into various derived products (chips, flour, cooked fermented pastes and fermented granular products such as *gari* or *atiéké*). Processing methods and product names differ from one region to another, and even within the same region. The diversity of cassava food uses is reflective of the cultural diversity of the producing populations. Fermented products are the major form of cassava consumed in almost all parts of Africa, accounting for almost 75% of cassava‐based foods (Westby, 1991). *Gari*, also called *garri*, *garry* or *tapioca*, depending on the producing area, is a toasted pregelatinised, fine to coarse granular flour, made from fermented cassava mash. It is the most traded and consumed cassava food product in West and Central Africa (Gouado *et al*., 2008; Sanni *et al*., 2009; Ngueulieu, 2013; Njukwe *et al*., 2013; Wassmer, 2013; Levai *et al*., 2016; Fon & Djoudji, 2017; Mapiemfu‐Lamaré *et al*., 2017; FAO, 2018a), which could be compared to what potato flour is to the Westerners. The growing popularity of gari as convenience food is mainly due to its affordability, easy storage and ease of preparation for consumption (Oluwafemi & Udeh, 2016). In Cameroon, consumption of gari is most common among people from the forest regions bordering Nigeria (Njukwe *et al*., 2013). Beyond the geographical proximity to Nigeria, which is the largest producer and consumer of gari, the common colonial heritage of these populations with Nigeria may justify the similarity of production and consumption of gari. Consumer trends and *gari* quality derived from the above studies have shown that taste (acid or sweet), colour (white or yellow) and grain characteristics (fineness and uniformity) account among the main attributes of gari on buying.

The high number of cassava varieties, with a diversity of quality characteristics, may lead to a large variability in the processing, use and quality of *gari*. The quality and acceptability of gari have been assessed in different studies with respect to cassava varieties (Tokula & Ekwe, 2006; Komolafe & Arawande, 2010; Sanoussi *et al*., 2015; Awoyale *et al*., 2020), coupled sometimes to area of production (Sanoussi *et al*., 2015; Olanrewaju & Oluwasola, 2017; Laya *et al*., 2018) and processing tools and practices (Olaoye *et al*., 2015; Tohnain & Bebnji, 2017). These studies focused on proximate composition, and functional and sensory properties of the gari. These studies provide an overview of the elements justifying consumers' needs in terms of product quality, thus highlighting the role of the cassava variety in the quality of gari. Identifying quality characteristics associated with cassava varieties and *gari* appears as a critical issue regarding the different processing practices, types of products, and processors’ and consumers’ expectations. Some of these quality characteristics can be linked to genetic traits and as such, integrated into breeding programs, leading to better adoption of new varieties. Varietal preferences start with the demand from a range of users/actors, such as producers, processors, retailers and consumers along the food chain. However, there is a gap in knowledge of preferences among different user groups, regarding the diversity of their needs, which may depend on how the crop is used and what products are made. This can result in multiple and, perhaps, contrasting preferences that vary according to the user’s role within the food chain, implying that the input and decision‐making roles of different users is of primary importance in crop breeding. Breeding programs have historically focused on yield and disease resistance to face the challenges of food security in terms of feeding a growing population (Ceballos *et al*., 2004; Manu‐Aduening *et al*., 2006; Ojulong *et al*., 2008; Ceballos *et al*., 2020), and on malnutrition and safety issues (Adenle *et al*., 2012; Peprah *et al*., 2020; Xing *et al*., 2020), with lower priority on post‐harvest quality characteristics, and processor and consumer demand. In addition, information on product characteristics is often overly simplified by not including information on the optimal range or description that would help breeder’s ability to meet user needs.

Often, farmers and processors will prefer earlier varieties, higher yielding and more dense/heavy (dry matter) roots but they will then assume that any new root will have the same quality of the varieties they are used to. Thiele *et al*. (2020) show, using data from a large cassava adoption study (Wossen *et al*., 2017), that in Nigeria, the largest area attributed to improved varieties is occupied by unreleased breeders’ material that are either the result of escaped clones from breeder trials or clones grown from botanic seed from released or unreleased material. This supports the case that it is not access to improved varieties that results into low adoption but makes a case for a large part of released varieties not living up to the quality characteristics assumed to be present in an ‘improved’ variety. This suggest that breeders should start evaluating their clones for characteristics preferred by end users along the food chain, through a dynamic process comparable to what has been done in heritability for starch and carotenoid composition of cassava (Olayide *et al*., 2020).

Fliedel *et al*. (2016) developed a new approach for providing better information to breeders early in varietal improvement programmes. It involved several successive steps, including qualitative surveys all along the food chain to identify quality criteria of a good cassava crop and product, and effective participation of processors to identify the ability of new genotypes to make a good product. Forsythe *et al*. (2021) adapted this approach in a multidisciplinary methodology to better understand end‐users’ demand of good quality root, tuber and banana (RTB) crop and products.

The aim of the present study, focused on *gari* in some major production and consumption areas of Nigeria and Cameroon, is to address these gaps through surveys on quality characteristics of cassava roots and processed product as perceived by users within the food chain, and contribute to shaping crop breeding to be more responsive to user needs.

## Materials and methods

### Survey locations and implementation

**Figure 1:**
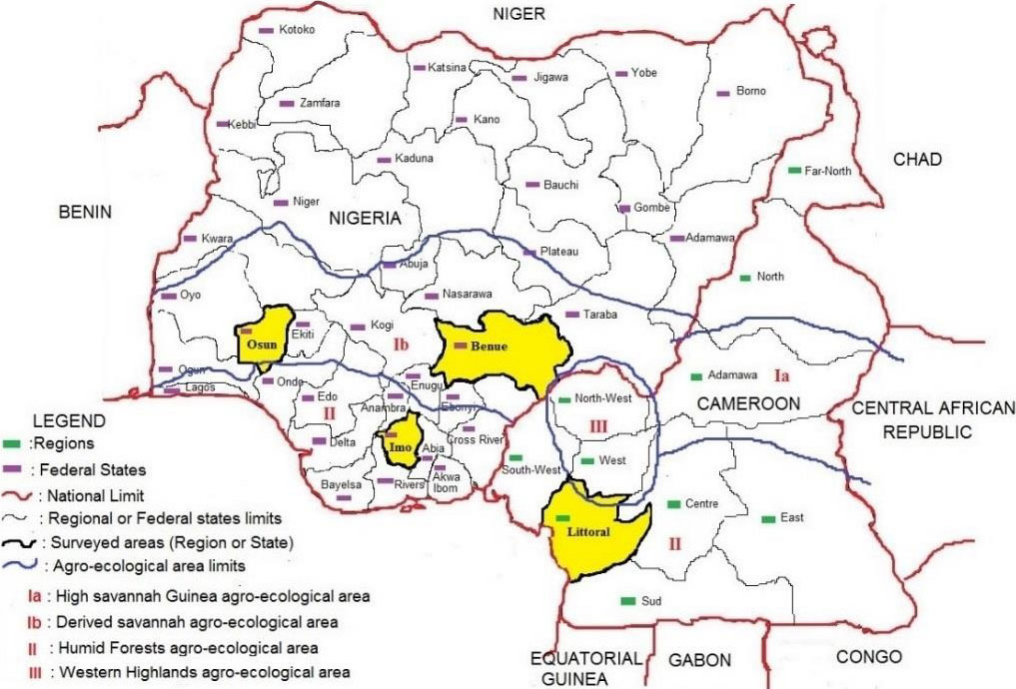
Survey *area (The States/Regions surveyed are highlighted in yellow).*

Our study was carried out between August 2018 and July 2019 in Cameroon (Littoral Region) and Nigeria (Imo, Osun, and Benue States; Fig. 1). In each area, interviews and discussion groups were carried out in at least four villages (Table 1) chosen on the basis of their current practices in cassava production and its processing into gari.

**Table 1:** Geographical references of survey areas with corresponding number of respondents

State/Region	Village	Latitude	Longitude	Number of respondents				
				KI	FGD	II	MI	Total per Region/ state
Littoral Region (Cameroon)	Bonagoum	4°14′6.87″N	9°36′56.7″E	6	17	10	4	131
	Bonamukandjo I	4°13′48.93″N	9°36′37.37″E	1	15	10	3	
	Passim	5°2′0.05″N	9°55′41.12″E	1	16	10	2	
	Nkongsoung Long Trait	5°7′36.42″N	9°57′38.97″E	1	6	0	0	
	Quartier 5	5°7′43.93″N	9°56′47.43″E	2	14	10	3	
Osun State (Nigeria)	Aba Gbooro Elefon	7°40′43″N	4°27′49″E	2	13	9	1	101
	Agoowu farm settlement	7°07′14″N	4°10′08.1″E	2	14	9	1	
	Oyan	8°02′40.7″N	4°02′16.7″E	2	12	10	1	
	Wasinmi Iseyin	7°26′7″N	4°15′52″E	2	13	9	1	
Benue State (Nigeria)	Tyomu	7°40′30.4″N	8°32′08.8″E	2	17	10	2	110
	Koti Shangev‐ya	7°40′38.5″N	8°32′15.2″E	2	12	9	0	
	Nyam II	7°40′37.2″N	8°32′14.0″E	2	15	9	1	
	Al′Okete	7°40′27.1″N	8°32′10.6″E	2	17	9	1	
Imo State (Nigeria)	Isinweke	5°38′34.16″N	7°21′45.14″E	1	17	10	1	124
	Amandugba	5°42′30.31″N	7°3′58.25″E	2	20	10	1	
	Akwakuma	5°48′32.29″N	7°3′39.35″E	1	21	10	1	
	Uzoagba	5°34′4.62″N	7°9′12.17″E	1	17	10	1	
		Totals		32	154	256	24	466

FGD, Focus Group Discussion; II, Individual Interview; KI, Key Informant; MI, Market Interview.

In each village, four target groups of stakeholders were interviewed according to Forsythe *et al*. (2021):

- Key Informant (KI) (at least 1 per village): a community leader, a person or a set of persons (1‐6 persons) who have a deep knowledge of the village. The interview provided information related to livelihood activities, social segmentation, varieties planted and utilisation of cassava in the village and contributed to sampling and probing in the next step of the interviews.
- Focus Group Discussions (FGD) (at least 2 per village, 1 for men & 1 for women): a group of 5 to 10 persons, sampled from information obtained from KI, who grow cassava and/or process it into *gari*. These groups were interviewed on questions related to farming practices, gender role, asset ownership, varieties planted (preference, utilisation and processing techniques), profile of ideal variety, preferences and characteristics (high and low quality) of cassava roots, intermediate products during processing and the final product *gari*, *gari* consumption forms and ingredients used for the preparation and/or consumption;
- Individual interviews (II): persons (10 persons at most – man or woman – per village), who farm cassava and process it into *gari*. They were interviewed on similar questions as in FGD, providing individual/household level description of preferred characteristics at different stages of product processing, household decision‐making and trade‐offs.
- Market interviews (MI) (at least 1 per village), made of a man or a woman, or a set of women who traded *gari*, and who were interviewed on aspects related to characteristics of sought *gari* and the profile of customers.

Altogether, 466 persons were interviewed in the 17 villages: 32 persons as KI (65.6% of men and 34.4% of women), 256 persons in FGD (52% of women and 48% of men), 154 persons as II (74% of women and 26% of men) and 24 persons in MI (19.2% of men and 80.8% of women). Before starting the interviews, explanations were supplied to the respondents about the objectives of the work, their importance as stakeholder in the achievement of these objectives, how the data would be used and data confidentiality. It is only when a given stakeholder consented to participate, and had signed the consent form, that the interview started. These interviews were carried out in the language that the respondents are fluent with.

### Data collection and analysis

Qualitative data collected from respondents (farmers and processors from FGD, processors from II and traders from MI) were analysed by coding and categorising information on quality characteristics (good and bad) of raw cassava roots and products (*gari* and its main consumed form, *eba*), using Multiple Criteria Decision Analysis (MCDA) approach, both at individual and location‐specific (State/Region) levels. At locality (State/Region) level, the analysis of ranked quality characteristics was conducted based on the methodology described by Forsythe *et al*. (2021) using pairwise ranking. Similar quality characteristics were grouped under a category term. The frequency of citation of each category term ranked as first, second or third is multiplied by 3, 2 and 1, respectively, and the values obtained for each category term summed. All the category terms of the locality were then ranked according to the obtained values, higher values corresponding to higher rankings, and weighted using the Ranking Ordered Centroid (ROC) method (Sureeyatanapas, 2016; Roszkowska, 2013; Tofallis, 2014; Sureeyatanapas, 2016). In order to bring out the main characteristics describing users’ demand, only the highest weight was used for diagrams.

Sphinx Plus^2^ – Edition Lexica‐V5, and Microsoft Excel software packages were used for treatment of questionnaires and analysis of data. Quality characteristics of cassava and gari were analysed based on their citation frequencies, and principal component analysis (PCA) was used to represent their distribution in the survey areas.

## Results and discussion

### Gender mapping of livelihood activities

Regardless of the area (Region/State), agriculture is the main livelihood activity, food crops and cash crops (coffee, palm oil trees and cocoa) being commonly farmed in all localities, with cassava as the major food crop farmed. Though in all localities, actors involved in agricultural activities originate from different national ethnic groups, or even countries, the ethnicity of actors is native dominated in Nigerian states (*Yoruba* in Osun State, *Tiv* in Benue State, and *Igbo* in Imo State), while in Littoral Region of Cameroon, *Bamilékés*, originating from Western Region of the country, constitute the majority of farmers, the native ethnic groups (*Abo* and *Mbo’o*) being in the minority.

The compilation of information obtained from KI and FGD indicates that gender mapping of farming systems is based on the wealth status of the actors, depending on the farmed surfaces, land ownership, crops, quantity harvested and level of work organisation. In this respect, wealthy farmers are mainly males, aged between 40 and 50 years old, who own land, farm food and cash crops intensively on high superficies (2–10 ha of cassava, 8–100 ha for cash crops), use important quantity of agricultural inputs, hire important number of labourers and use agricultural machinery (in Nigeria only). Wealthy farmers represent about 10% of Nigerian farmers, with men representing double the number of wealthy women. In all areas, poor farmers are mainly females, representing more than 80% of agricultural actors, who do not have secure land tenure, farm numerous food crops at small scale and on low surface (1/4 to 2 ha for cassava), and cannot afford important quantity of agricultural inputs. These findings are comparable in almost all the study area and are perceived in the same proportion from KI and FGD. Land cultivation is generally done in mounds and in ridges in almost all areas. Meanwhile, when soil is clayey and soft, as in Imo State, farming in flat soil is practiced. From gender point of view concerning farming practices, locality specificity is observed. In Benue state, men generally farm in mounds and women in ridges, while in Osun State and Littoral Region of Cameroon, farming in ridges is more common, both for men and women. Mono‐ and mixed‐cropping are practiced in all areas, mono‐cropping being more common in Nigeria and done by wealthy farmers, who use tractors, while in Cameroon, the practice is scarce and only found among specific crops (tomatoes, pineapple, palm oil trees) for high national, regional or international markets. Mixed‐cropping is generally more often practiced by women who combine subsistence needs and household chores with farming work by mixing crops of long vegetation cycle (cassava, yam and cocoyam) with those of shorter vegetation cycle (maize and groundnuts). This mixed‐crops practice is mainly for home consumption orientation farming and constitutes a way to maximise use of land, since land availability and land ownership appear among the major constraints of agricultural activities (Kébé Diouf, 2016; Nkuintchua, 2016). In this respect, most smallholder farmers in Cameroon rent land on which they farm. In areas where land is available, as in some localities of Osun State, men and women work on separate farms. When land is scarce, men and women work in the same farm, but generally on separate plots, many food crops being found on women’s plots and used both for market and for home consumption. In Nigeria, immigrants from other Nigerian states and countries (mainly Benin and Togo) in Osun State tend to work together on the same land with less pronounced gender division of labour (Forsythe *et al*., 2016). Regardless of the availability of land, farming tasks are shared when a couple is working on the same land. In this respect, men are involved, to a greater extent, in farm preparation (clearing of the farm, spraying of herbicides, digging of holes for mounds), while women are more involved in tasks which require patience with less physical strength (planting, weeding and harvesting). Some crops are specific to men in some localities (tomatoes in Littoral region of Cameroon and yam in Imo State, Nigeria). The investment cost (case of yam) and the risk level (high perishability and variability of market demand for tomato) associated with the crop justify this differentiation. Men are more willing to take risks while women, because of their involvement in many more household related tasks besides farming, are less inclined to take risks likely to hamper the family care and food supply. Studies have shown that men start to grow or even take over production of crops previously dominated by women when they become more productive or profitable (Doss, 2001; Whitehead*et al*., 2000; World Bank *et al*., 2009; Fischer & Qaim, 2012; David, 2015). Regardless of the location, cassava is either the most farmed or among the most farmed crops, and the majority of harvested roots are processed. The proportion of cassava used for household consumption is higher in Nigeria (generally around 40%) compared to Cameroon (generally around 20%), confirming the importance of cassava in the diet of Nigerians. In the South‐West of Nigeria, processing of cassava into fermented products is mainly carried out by women using the service of processing centres that offer grating, pressing and toasting facilities, while in Imo and Benue state as well as the littoral zone in Cameroon, processing takes more often place within the household, using own equipment (or using a mobile grater service that goes from compound to compound), in which relatively more men participate. However, one major and important finding which confirms earlier findings (Curran *et al*., 2009; Walker *et al*., 2014; Njukwe *et al*., 2014; Taiwo & Fasoyiro, 2015; Fon & Djoudji, 2017; Teeken *et al*., 2018) is the overall dominance of women within the processing of cassava into food products as well as the marketing of these products in all the regions. Given the disadvantaged position of women found in our study, more attention for processing and food quality related cassava traits within the development of new varieties will therefore benefit many women in Cameroon and Nigeria.

### Cassava varieties and their preferred characteristics

**Figure 2:**
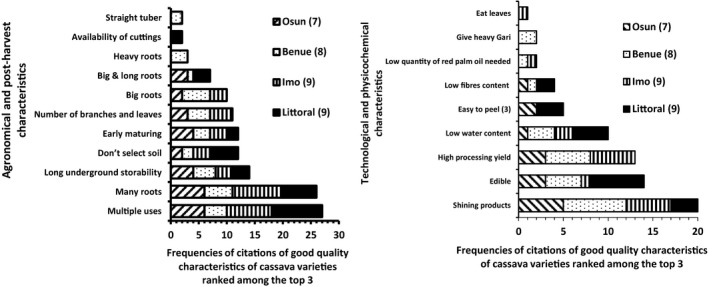
High‐quality characteristics of top 3 cultivated cassava varieties cited by farmers during FGD and II (For each locality, the number in the parenthesis represent the number of top 3 liked varieties, all the villages being considered).

Different cassava varieties, with names varying from one farmer to another, or by village, are farmed in all areas, depending on food habit, derived cassava food product, market environment and other. The differentiation of these varieties is based both on agronomical and post‐harvest (processing and consumption) quality characteristics of the roots. Major characteristics of the top three varieties farmed in each area are shown in Fig. 2. The preferences of specific root quality characteristics are similar in the study areas, which confirms information currently reported from other surveys carried out in SSA countries (Agbor‐Egbe & Lape Mbome, 2006; Zundel *et al*., 2010; Njukwe *et al*., 2013; Ukenye *et al*., 2013; Mouafor *et al*., 2016; Wossen *et al*., 2017; Teeken *et al*., 2018). At farming stage, yield and suitability for multiple end uses are the major characteristics cited by respondents, followed by other agronomical and post‐harvest characteristics such as size of roots, storage ability in soil after maturity, adaptation to poor soils and early maturity. At processing and consumption stages, the root should also give shining/attractive products, including *gari*; be edible (sweet taste and good cooking quality, which facilitate fresh consumption); have high processing yield (low water content, and ease of peeling); and low fibre content – in a decreasing order of importance based on number of citations. In Nigeria, the preference of cassava varieties with a good number of branches and leaves, or providing good canopy, is, according to respondents (II & FGD), related to the aptitude of these varieties to suppress weeds and to provide sufficient stems for the next farming season. There is also the overall common belief that cassava plants with a good canopy always have a good yield.

**Figure 3:**
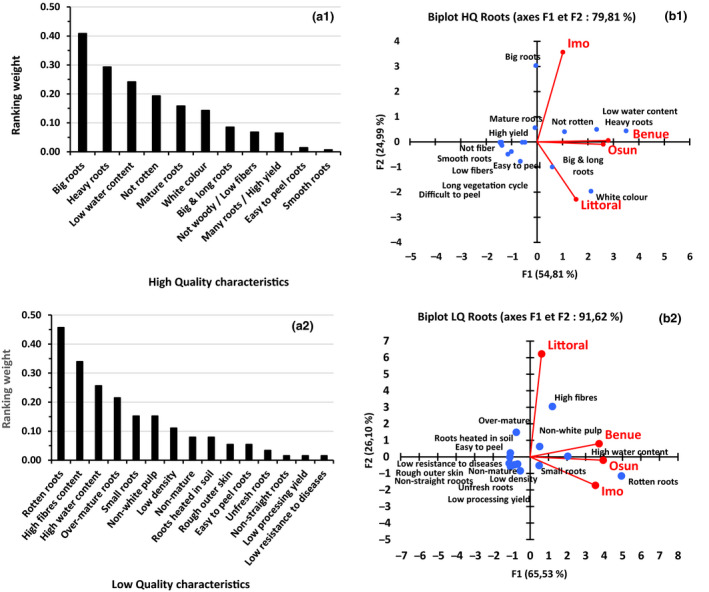
Weight distribution of high (a1)‐ and low (a2)‐quality characteristics of cassava roots and their representation in producing areas (b1, b2).

When grouping and ranking the importance of specific cassava root characteristics in general, regardless of the varieties (Fig. 3), the characteristics oriented towards processing and end‐use issues – for example size, density, water content, safety (not rotten) and colour – indicate farmer perceptions of high quality. Though the type of quality characteristics, both high and low, is common to all producing areas, their importance can vary from one region to the other (Fig. 3b1 & b2). On the other hand, there are sometimes high similarities between areas, such as Benue and Osun States for high‐quality characteristics, and in Benue, Osun and Imo States for low‐quality characteristics. Areas can show strong differences from one another, based on the difference in weighting attributed to a specific characteristic, depending either on local specific farming conditions or on end‐use orientation of the root. This is the case in Imo State and Littoral Region, for which the major high‐quality characteristics, with far higher weight, are, respectively, big root size and white colour of the roots (Fig. 3b1). In the same vein, the high rejection of high fibre content of roots in Littoral Region distinguishes this area from the others in terms of varieties that will be grown (Fig. 3b2). The importance of big roots in Imo State can be understood for three reasons: (i) bigger roots reduce the total surface that has to be peeled off, which is convenient for women, the main actors involved in peeling; (ii) there is land scarcity in Imo and the average size of cassava plots is lower than in most other places (Korieh, 2010) because of lineage dividing up communal land with growing population, making optimising production yield relatively more important as increasing yields per area are the only way to obtain more roots; *iii*) soils in Imo are relatively acid in comparison with the other regions making less nutrients available to the plant resulting in relatively smaller roots.

**Figure 4:**
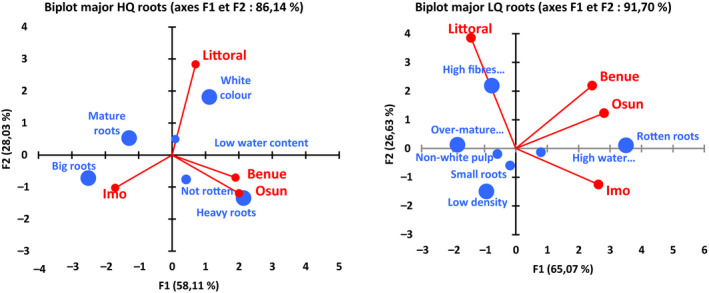
Distribution of major quality characteristics (high and low) of cassava roots in producing areas (the size of a characteristic’s marker is representative of its relative weight).

Considering the distribution of quality characteristics of roots in the producing areas (Fig. 3.b1 & b2), it appears that all characteristics displaying a ranking weight ≤ 0.1 are localised around the central point of the PCA graph. This may indicate the relative low impact of these characteristics. Thus, all root quality characteristics (high and low) with ranking weight ≥ 0.1 can be considered as more representative of farmers’ perceptions. With regard to this assertion, the PCA representation of major quality characteristics of roots in all areas (Fig. 4) indicates that high‐quality cassava roots are mainly determined by the following characteristics: size (‘big roots’, ‘big and long roots’); density (‘heavy roots’); ‘low water content’, which could be coupled to ‘density’ and its ‘safe character’ (‘not rotten’); maturity (‘mature roots’); and colour (‘white colour’). On the other hand, low‐quality root is mainly characterised by its unsafe character (‘Rotten root’); its fibre and water contents (‘high fibre content’ and ‘high‐water content’); its maturity stage (‘over‐mature root’); its size and density (‘small size’ and ‘low density’); and the colour of its pulp (‘non‐white pulp’). Farmers consider these characteristics in choosing which varieties to cultivate. The decision is also related to end use of the roots, in particular which type of products are to be processed. In this respect, the preferred characteristics of the end‐product (*gari* in our case) should be known. Bechoff *et al*. (2018) indicated that diversity in cassava food products can provide a challenge to identifying acceptance criteria, and socio‐economic factors such as gender may also be critical. This is the case, for instance, for the colour of cassava pulp for gari. In Littoral Region of Cameroon, the end use of the root orients the preference of cassava varieties with white pulp since cassava varieties with yellow pulp are used exclusively for home consumption, and rarely for market; in addition, yellow varieties are still found in very limited areas. In Nigeria, yellow varieties are more common and sometimes used for gari processing, since no additional oil is required to obtain a yellow *gari*. In the same vein, the higher ranking of high fibre content of roots among low characteristics of cassava roots in Littoral Region may be related to farming practice in this area where root varieties with long vegetative cycle are used, which results in an increase in lignification. Ease of peel also appears among the high and low preference characteristics. This might be attributed to the fact that such roots have a relatively high‐water content and thus a rather low yield in processing. Processors could therefore notice that a root that is easy to peel would indicate low dry matter content, which will result in lower food product yield.

The determinants associated by farmers to the quality characteristics of roots raise scientific issues that need further exploration. The above high‐ and low‐quality characteristics of cassava roots are often displayed in sub‐optimal combinations in currently cultivated varieties, that is no varieties show only high‐quality characteristics, without one or more low‐quality ones. For example, early maturing varieties are, in general, negatively characterised by high‐water content of roots, resulting in low storability in the soil beyond maturity. But on the positive side, early maturing varieties tend to display good yield in low fertility soils, easy removal of peels and low fibre content (Teeken *et al*., 2018). On the contrary, late maturing varieties display positively, low water content, high processing yield and storability in soil beyond maturity, but negatively display high yielding only on fertile soils, difficulty of peeling and high fibre content. Growers are faced with the difficult decision of choosing a balance between positive and negative characteristics that best meet their needs. This constraint leads the farmers to adapt their choice to their technical and environmental possibilities. Thus, when an early maturing cassava variety is planted, it must not be harvested too early or late, provided that the farmer has the technical means to harvest the entire production at once. On the contrary, farming of late maturing cassava varieties allows piecemeal harvesting, in line with processing capacity and market demand. Farmers often plant both types of varieties using the short cycle varieties as hunger breakers, allowing cassava to be available for a longer stretch of time, facilitating continuous processing and thus income generation. Sanginga (2015) noted that yam and cassava, though longer in their cropping cycle, are vital in the annual cycle of food availability due to their broader agroecological adaptation, diverse maturity period and in‐ground storage capability, permitting flexibility in harvesting period for sustained food availability.

All the quality characteristics of cassava were mentioned as preferences by both men and women, regardless of the area, except that women attribute higher weight than men to ease of peeling. This is likely because peeling is generally the responsibility of women, with help of children.

### Quality characteristics of gari

#### Cassava processing into gari

**Figure 5:**
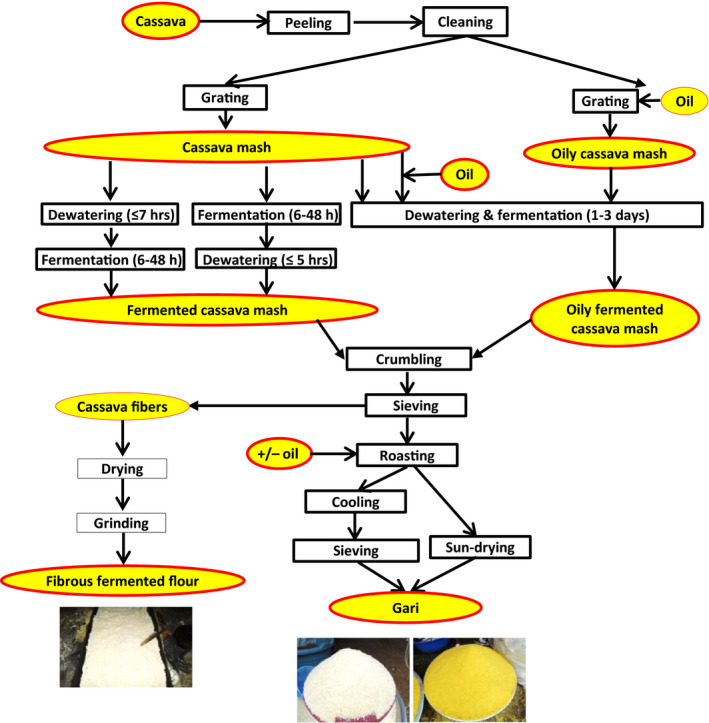
Variants of cassava processing into gari.

Cassava processing into gari involves successive unit operations including: peeling, washing, grating, dewatering to obtain a mash, fermenting, crumbling, sieving and finally toasting to obtain the pregelatinised granulated *gari*. Different variants are used by processors in setting up the unit operations, in terms of ordering and processing time (Fig. 5). To produce yellow *gari*, palm oil is added at different steps of processing, for example after grating and before mash fermentation, or during toasting of the sieved fermented mash. The specific step depends on processors’ practice, objective and area. For instance, gari processors in the Littoral Region of Cameroon assert that adding palm oil before fermentation facilitates the toasting and produces *gari* with homogeneous colour and no lumps. The yellow *gari* is highly predominant in Cameroonian markets, while in Nigeria, yellow gari, processed either using palm oil or using yellow cassava varieties only, is predominant in markets of South‐East and North‐Central States (including Imo and Benue States, respectively); on the contrary, white *gari* is more abundant in markets of South‐West States (including Osun State) (Udofia *et al*., 2011; Adebayo *et al*., 2012; Funke *et al*., 2012). In some *gari*‐producing areas, residues, such as fibres resulting from sieving (Fig. 5), can be value‐added by drying (sun or firewood) and grinding to obtain a fibrous fermented flour used to prepare a dough called *Kumkum* in Cameroon. Most of the variants of cassava processing into *gari* in different African countries were reported in the literature (Afoakwa *et al*., 2010; Adebayo *et al*., 2012; Ukpabi *et al*., 2012; Onasoga *et al*., 2014; De Moura *et al*., 2015; Ikpe & Essienubong, 2016; Fon & Djoudji, 2017; Olanrewaju & Idowu, 2017; FAO, 2018b; Adinsi *et al*., 2019), showing spatial variability of processing and quality of *gari*.

#### Quality characteristics of gari

**Figure 6:**
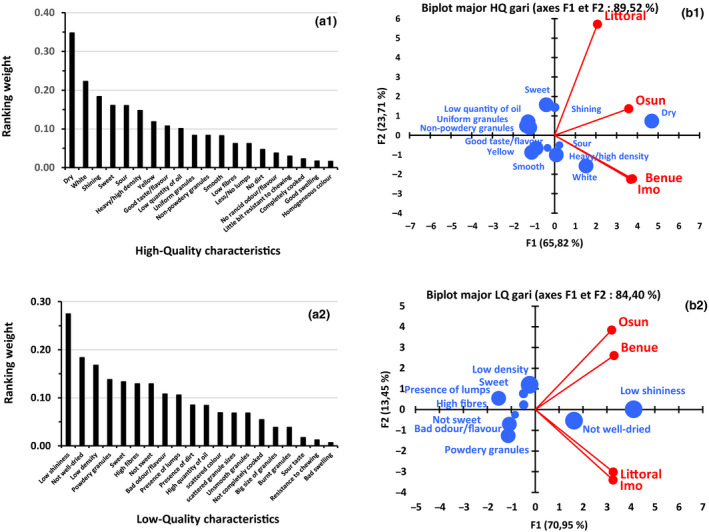
Weight distribution of major high (a1)‐ and low (a2)‐quality characteristics of gari cited by processors and their representation in the different areas in Nigeria and Cameroon (b1, b2) (size of characteristics’ markers in PCA are related to their weight).

Important high‐ and low‐quality characteristics of *gari* in the different survey areas, presented in Fig. 6, show that good gari is mainly characterised by its appearance (shiny, smooth, uniform and non‐powdery granules, low level of fibre, no lumps and white or yellow colour), its texture in hand and mouth (high density, dry, a bit resistant to chewing, good swelling), its taste (sweet, sour) and its aroma and flavour. Depending on individual preferences and locality, opposite quality characteristics can be displayed in the description of a good quality *gari*. For example, sweet taste and sour taste both appear among the high‐quality characteristics cited by respondents, though with different weights. The weights of the different *gari* quality characteristics vary between localities and can be supported by cultural determinants, according to Agbor‐Egbe and Lape Mbome (2006) and Levai *et al*. (2016). French speaking consumers in Cameroon generally prefer yellow and little bit sweet *gari*, similar to the *Idoma* population in Nigeria (Benue State, North‐Central Nigeria). On the contrary, English‐speaking consumers in Cameroon (North‐West and South‐West Regions) prefer white and strongly sour *gari*, similar to the *Yoruba* population of South‐West Nigeria, including Osun State and *Igbo* population of South‐East Nigeria, including Imo State. However, the *Yoruba gari* is sourer than that from other regions, and the *Igbo* population produces yellow as well as white gari. Preferences for sweet gari and less sour one are generally observed for ethnic groups which do not have gari as a cultural main course meal (French speaking ethnic groups of Cameroon and *Idoma* ethnic groups of Nigeria). Moreover, the territorial proximity and continuity of English‐speaking Cameroonians, *Ibo* and *Yoruba* (Fig. 1) may constitute another explanation.

The quality characteristics of gari depend both on the cassava variety and on the process used, the latter having the major influence (Fig. 6). However, since poor cassava quality may lead to acceptable gari, subject to suitable production process, the relationship between cassava quality characteristics and processing practice is important in determining gari quality. The end use of gari is another factor to be taken into account.

#### Quality characteristics of eba, a traditional paste made from gari

*Gari* is consumed in different forms (Table 2). The most popular ones, regardless of the area, are *gari* added with water and eaten after soaking (soaked *gari*) and *gari* cooked into a paste (*eba*). These two consumption forms take advantage of the swelling properties of *gari*. *Gari* added with cold water, sugar and/or other ingredients is consumed as a snack, mainly by young people or students between meals, while *gari* cooked into a paste, popularly called *eba* in Nigeria and *gari fu*fu/*couscous tapioca*/*couscous gari* in Cameroon, is generally eaten with a soup as part of a family meal. In this paper, we will use the term *eba*.

**Table 2:** Gari consumption forms

Consumption form	Preparation	Country
Soaked Gari	Soaking in cold water, addition of sugar and eating with different ingredients (groundnuts, coconuts, beans, milk, etc.)	Nigeria/Cameroon
Eba/Gari fufu/Couscous gari	Pouring gari into boiled water and cooking under stirring for 2–5 min	
	Mixing of boiled water and Gari and turned to paste without cooking	
Fried gari/Gari sauté/	Frying of gari with oil and spices, and sometimes with eggs, then eating as such	Cameroon
Omelette Tapioca	Frying of a mixture of crude eggs, Gari and flavors in oil	
Purée de Tapioca	Puree obtained from a mixture of gari and avocado	

**Figure 7:**
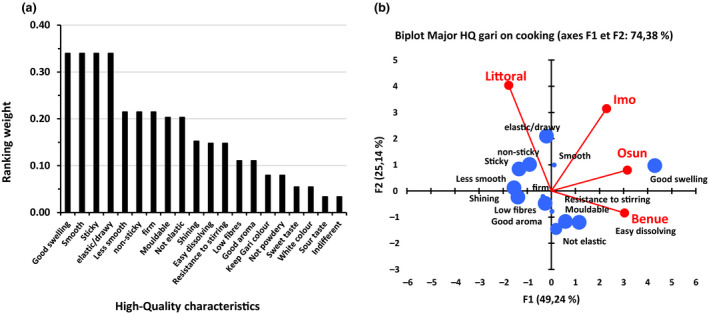
Weight distribution of major high‐quality characteristics of gari on cooking (a) and their representation in the different areas in Nigeria and Cameroon (b) (size of characteristics’ markers in PCA are relative to their weight).

**Figure 8:**
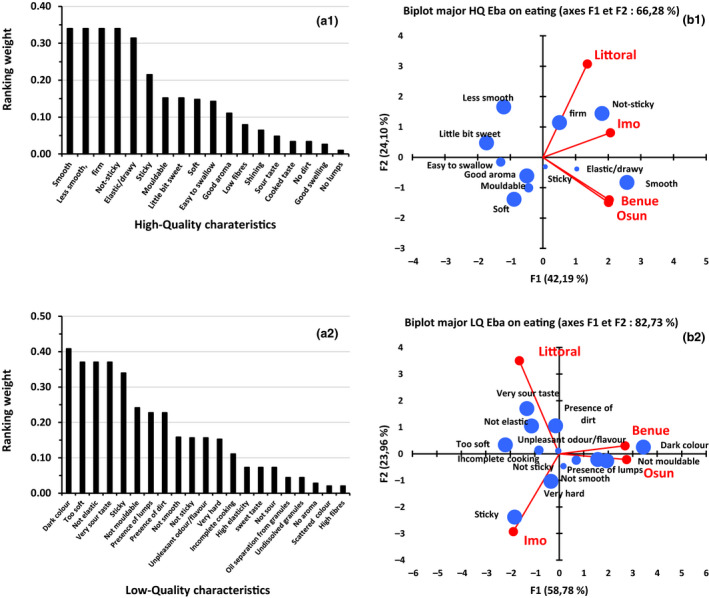
Weight distribution of high (a1)‐ and low (a2)‐quality characteristics of eba ready to eat and their representation in the different areas in Nigeria and Cameroon (b1, b2) (size of characteristics’ markers in PCA are relative to their weight).

The high‐quality characteristics of *eba* are related to its behaviour during cooking (good swelling) (Fig. 7), and its texture from cooking to eating stage such as easy to swallow, smooth, sticky, not sticky, elastic/drawy, soft, firm and mouldable (Fig. 8). High‐quality characteristics of *eba* are also related to its appearance (shiny, no lumps, no dirt), its taste (little bit sweet, sour, cooked taste) and aroma. On the opposite side of the quality spectrum, a bad quality *eba* is characterised by its appearance (presence of lumps, of dirt and dark colour), its sour taste, its flavour (unpleasant odour/flavour, incomplete cooking) and its texture (not smooth, sticky, not elastic and not mouldable).

Based on the terms used by respondents, the texture characteristics of *eba* may be related to quality characteristics of *gari* such as granule uniformity, ease of dissolving, colour, level of powderiness and resistance to stirring. These quality characteristics of the *gari* may impact on the swelling, smoothness, stickiness, elasticity and mouldability properties of *eba*. As in the case for *gari* taste, respondents cited opposite quality characteristics (mainly the texture) as the preferred ones for *eba*. Therefore, *eba* is liked either soft, a little bit sticky, smooth and elastic, or firm, not sticky, less smooth and less elastic. This differentiation seems to be specific to locality/ethnicity and cooking process, since *eba* is appreciated either for the strong or weak behaviour of its texture (Fig. 8). Especially in Osun and Benue States, dark colour of eba is a major indicator of low quality, while this is less so for Imo and Littoral, while low shininess (low brightness) is an important low‐quality gari characteristic for all the regions. This non‐preference might be related to the colouring of the *gari* with palm oil which is more frequent in Imo and Littoral than it is in Osun and Benue, probably making a darker colour less attractive. It might also be related to the longer fermentation time practiced in Osun and Benue relative to Littoral and Imo which might create higher coincidences of darkening when *gari* is turned to *eba*. Also, sun drying of *gari* is known to be practiced in Osun state and this might result into discolouring through contamination with dust and or reaction with fungi/bacteria, a discoloration that can be amplified when turning the dry *gari* product into a wet *eba* product.

The prominence of ‘not mouldable’ as a low‐quality eba characteristic in Benue and Osun States is related to the mode of preparation: in both Benue and Osun, *eba* is prepared by mixing of boiled water and *gari* and turning into a paste with a spoon. This allows for less homogeneous rehydration to take place and providing the *eba* with a relatively crumblier and more airy structure. This method naturally creates more coincidences of mouldability problems (depending on the structure of starch in *gari*) as the *eba* can fall apart (crumble) more easily if starch gets less time to rehydrate and gelatinise compared to *eba* that is prepared by cooking, which results in a more homogenous rehydration where the *gari* particles are better merged because of the longer contact between hot water and *gari*.

## Conclusions

Maturity, size and density of cassava roots constitute the main high‐quality characteristics of raw cassava cited by farmers in the study areas in Nigeria and Cameroon. For *gari*, a toasted fermented granular product, the main high‐quality characteristics identified by respondents, concern mainly the appearance, the taste, the dryness and density of the product, while for *eba*, (a cooked paste made from *gari*), users identified textural characteristics as most important. Processors use almost all cassava varieties available in their area for making *gari* through different process variants, implying that cassava root of low quality may be processed into acceptable *gari*. This might indicate the significant role of processing techniques in determining the quality of *gari* and of its derivative products, raising a logical issue on the importance of the relationship between cassava roots characteristics, processing techniques, and *gari* and *eba* quality, in line with the preferences of end users. It should then be useful to work with processors through a participatory approach, to identify with them the key steps in the processing that influence the *gari* quality and the *eba* quality (using very different cassava varieties, adapted or non‐adapted for making a high‐quality product). Consumers’ overall liking of these products will be related to their different sensory properties and other quality characteristics.

It is often at the moment of the introduction of newly bred varieties that farmers first encounter roots that do not provide an optimal product. Looking at the results of this study for high‐quality characteristics for fresh roots, it is easy for breeders to see their current selection criteria confirmed. However, the difficultly with characteristics such as yield, early maturity and high dry matter is that they are so called quantitative ‘empty traits’ not considering quality trade‐offs.

The results of this study raise an issue on a dynamic cooperation process between breeders and food scientists to investigate proof of concepts on the relation between physicochemical traits in fresh roots and food product quality, in order to connect physicochemical properties to genetic markers. It also involves testing of clones and cooperation with most often women processors who are the custodians of detailed processing expertise and knowledge of cassava food product quality. Women will also be important beneficiaries of these proposed strategies given their disadvantaged position within many communities and their prominent role in cassava processing and marketing of gari.

## Author Contribution

**Robert Ndjouenkeu:** Conceptualization (lead); Data curation (equal); Formal analysis (equal); Investigation (equal); Writing‐original draft (lead); Writing‐review & editing (equal). **Franklin Ngoualem Kegah:** Data curation (equal); Formal analysis (equal); Investigation (lead); Writing‐original draft (equal). **Béla Teeken:** Conceptualization (equal); Data curation (equal); Formal analysis (equal); Writing‐review & editing (equal). **Benjamin Okoye:** Writing‐review & editing (equal). **Tessy Madu:** Data curation (equal); Formal analysis (equal); Writing‐review & editing (equal). **Olamide Deborah Olaosebikan Olamide Olaosebikan:** Data curation (equal); Formal analysis (equal); Writing‐review & editing (equal). **Ugo Chijioke:** Writing‐review & editing (equal). **Bello Abolore:** Writing‐review & editing (equal). **Adebowale Oluwaseun Osunbade:** Writing‐review & editing (equal). **Durodola Owoade:** Writing‐review & editing (equal). **Noel Hubert Takam‐Tchuente:** Data curation (equal); Investigation (equal). **Esther Biaton Njeufa:** Data curation (equal); Investigation (equal). **Isabelle Linda Nguiadem Chomdom:** Data curation (equal); Investigation (equal). **Lora Forsythe:** Methodology (lead); Writing‐review & editing (equal). **Busie Maziya‐Dixon:** Writing‐review & editing (supporting). **Genevieve Fliedel:** Methodology (equal); Writing‐review & editing (equal).

## Conflict of interest

The authors declare no conflict of interest in this work.

## Ethical guidelines

The article is part of a project approved by Cameroonian and Nigerian authorities in the framework of RTB development and implemented by international and national institutions based in these countries. IITA has the mandate to carry out research in Nigeria including human subjects. Participants were informed about the study, they could stop the interview at any point, written consent from sensory panellists and from consumers participating in this study were obtained and the research respected the rules of voluntary participation and anonymity.

### Peer Review

The peer review history for this article is available at https://publons.com/publon/10.1111/ijfs.14790.

## Data Availability

The data that support the findings of this study are available from the corresponding author upon reasonable request.

## References

[ijfs14790-bib-0001] Adebayo, B.A. , Nanam, T.D. , Bamidele, E.A. & Braima, D.J. (2012). Quality Management Manual for the Production of Gari. Ibadan, Nigeria: International Institute of Tropical Agriculture. http://hqcf.iita.org/wp‐content/uploads/2016/04/7_quality‐manangmeny‐for‐gari‐production.pdf.

[ijfs14790-bib-0002] Adenle, A.A. , Aworh, O.C. , Akromah, R. & Parayil, G. (2012). Developing GM super cassava for improved health and food security: future challenges in Africa. Agriculture & Food Security, 1, 11.

[ijfs14790-bib-0003] Adinsi, L. , Akissoé, N. , Escobar, A. *et al*. (2019). Sensory and physicochemical profiling of traditional and enriched *gari* in Benin. Food Science & Nutrition, 7, 3338–3348. 31660147 10.1002/fsn3.1201 PMC6804918

[ijfs14790-bib-0004] Afoakwa, E.O. , Kongor, E.J. , Annor, G.A. & Adjonu, R. (2010). Acidification and starch behaviour during co‐fermentation of cassava (Manihot esculenta Crantz) and soybean (Glycine max Merr) into gari, an African fermented food. International Journal of Food Sciences and Nutrition, 61, 449–462. 20109125 10.3109/09637480903393727

[ijfs14790-bib-0005] Agbor‐Egbe, T. & Lape Mbome, I. (2006). The effects of processing techniques in reducing cyanogen levels during the production of some Cameroonian cassava foods. Journal of Food Composition and Analysis, 19, 354–363.

[ijfs14790-bib-0006] Awoyale, W. , Asiedu, R. , Kawalawu, W.K. *et al*. (2020). Assessment of the suitability of different cassava varieties for gari and fufu flour production in Liberia. Asian Food Science Journal, 14, 36–52.

[ijfs14790-bib-0007] Bechoff, A. , Tomlins, K. , Fliedel, G. *et al*. (2018). Cassava traits and end‐user preference: Relating traits to consumer liking, sensory perception, and genetics. Critical Reviews in Food Science and Nutrition, 58, 547–567. 27494196 10.1080/10408398.2016.1202888

[ijfs14790-bib-0008] Ceballos, H. , Iglesias, C.A. , Perez, J.C. , Alfred, G.O. , and Dixon, A.G.O. (2004). Cassava breeding: opportunities and challenges. Plant Molecular Biology, 56, 503–516. 15630615 10.1007/s11103-004-5010-5

[ijfs14790-bib-0009] Ceballos, H. , Rojanaridpiched, C. , Phumichai, C. *et al*. (2020). Excellence in cassava breeding: perspectives for the future. Crop Breeding, Genetics and Genomics, 2, e200008.

[ijfs14790-bib-0010] Curran, S. , Leigh‐Anderson, C. , Gugerty, M.K. , Cook, J. , Yorgey, G. & Gockel, R. (2009). Gender and cropping: Cassava in Sub‐Saharan Africa. Evans School Policy Analysis and Research (EPAR) brief no. 223. Prepared for the Agricultural Policy and Statistics Division of the Bill and Melinda Gates Foundation. https://evans.uw.edu/sites/default/files/Evans_UW_Request%2032_Gender%20and%20Cropping_Cassava_05‐20‐2009.pdf.

[ijfs14790-bib-0011] David, S. (2015). Getting a piece of the pie: An analysis of factors influencing women’s production of sweet potato in Northern Nigeria. Journal of Gender, Agriculture and Food Security, 1, 1–19.

[ijfs14790-bib-0012] De Moura, F.F. , Miloff, A. & Boy, E. (2015). Retention of provitamin A carotenoids in staple crops targeted for biofortification in Africa: cassava, maize, and sweet Potato. Critical Reviews in Food Science & Nutrition, 55, 1246–1269. 24915386 10.1080/10408398.2012.724477 PMC4353306

[ijfs14790-bib-0013] Doss, C. (2001). Designing agricultural technology for African women farmers: lessons from 25 years of experience. World Development, 29, 2075–2092.

[ijfs14790-bib-0014] FAO (1991). Racines, tubercules, plantains et bananes dans la nutrition humaine. ISBN 92‐5‐202862‐5. FAO, Rome. http://www.fao.org/3/t0207f/T0207F00.htm#Contents.

[ijfs14790-bib-0015] FAO (2018a). Commodities by country. Food and Agriculture Organization of United Nations. http://www.fao.org/faostat/en/#rankings/commodities_by_country.

[ijfs14790-bib-0016] FAO . (2018b). Étude Diagnostique de la réduction des pertes après récolte de trois cultures: manioc – tomate – pomme de terre. Cameroun. Rome: Rapport de synthèse.

[ijfs14790-bib-0017] Fischer, E. & Qaim, M. (2012). Gender, agricultural commercialization, and collective action in Kenya. Food Security, 4, 441–453.

[ijfs14790-bib-0018] Fliedel, G. , Monteiro, M.J. , Tomlins, K. I. *et al*. (2016). New approach for better assessing consumer acceptability of improved cassava food products. In: Electronic Proceedings of First World Congress on Root and Tuber Crops WCRTC, Nanning, China, 18/01/2016 to 22/01/2016. http://www.gcp21.org/wcrtc/S20.html.

[ijfs14790-bib-0019] Fon, E.D. & Djoudji, S.T. (2017). Potentials for cassava processing in the Littoral region of Cameroon. International Journal of Agricultural Economics, 2, 122–128.

[ijfs14790-bib-0020] Forsythe, L. , Posthumus, H. & Adrienne, M. (2016). A crop of one's own? Women’s experiences of cassava commercialization in Nigeria and Malawi. Journal of Gender, Agriculture and Food Security, 1, 110–128.

[ijfs14790-bib-0021] Forsythe, L. , Tufan, H. , Bouniol, A. , Kleih, U. & Fliedel, G. (2021). An interdisciplinary and participatory methodology to improve user acceptability of root, tuber and banana varieties . International Journal of Food Science and technology; Special Issue: Consumers have their say: assessing preferred quality traits of roots, tubers and cooking bananas, and implications for breeding, 56, 1115–1123. 10.1111/ijfs.14680 PMC7984279 33776224

[ijfs14790-bib-0022] Funke, O. , Raphel, B. & Kabir, S. (2012). Market structure, conduct and performance of *gari* processing industry in South Western Nigeria. European Journal of Business and Management, 4, 99–113.

[ijfs14790-bib-0023] Gouado, I. , Mawamba, D.A. , Ouambo, M.R.S. , Some, T.I. & Tchouanguep, M.F. (2008). Provitamin A carotenoid content of dried fermented cassava flour: the effect of palm oil addition during processing. International Journal of Food Engineering, 4(4), , 10.2202/1556-3758.1167.

[ijfs14790-bib-0024] IFAD (International Fund for Agricultural Development) . (2008). Etude sur les potentialités de commercialisation des produits dérivés du manioc sur les marchés CEMAC. https://www.doc‐developpement‐durable.org/file/Gestion‐entreprises‐et‐associations/commercialisation/Acces%20au%20marche%20&%20commercialisation%20de%20produits%20derives%20du%20manioc.pdf

[ijfs14790-bib-0025] Ikpe, N.E. & Essienubong, I.A. (2016). Optimization of indigenous food (*Gari*) fermentation with respect to time and texture. International Journal of Innovative Science, Engineering & Technology, 3, 98–107.

[ijfs14790-bib-0027] Kébé Diouf, K. (2016). Genre et foncier : l’expérience des consultations juridiques gratuites au Sénégal. London: IIED 18 p. https://www.iisd.org/sites/default/files/meterial/session‐1‐hubert‐ouedraogo‐contexte%20foncieres‐fr.pdf.

[ijfs14790-bib-0028] Komolafe, E.A. & Arawande, J.O. (2010). Evaluation of the quantity and quality of gari produced from three cultivars of cassava. Journal of research in national development, 8, https://www.transcampus.org/JORINDV8Jun2010/JournalsV8NO1Jun201039.htm.

[ijfs14790-bib-0029] Korieh, C.J. (2010). The Land has Changed: History, Society and Gender in Colonial Eastern Nigeria. Calgary: University of Calgary Press.

[ijfs14790-bib-0030] Laya, A. , Bargui Koubala, B. , Kouninki, H. & Nchiwan Nukenine, E. (2018). Effect of harvest period on the proximate composition and functional and sensory properties of gari produced from local and improved cassava (Manihot esculenta) varieties. International Journal of Food Science, 2018, 6241035. 10.1155/2018/6241035 29850481 PMC5907523

[ijfs14790-bib-0031] Levai, L.D. , Nsimi, M.A. , Nana, G. *et al*. (2016). Consumer perception of *Gari* prototypes and prospects for improvement and marketing in the South West region of Cameroon. International Journal of Agriculture and Environmental Research, 2, 1304–1318.

[ijfs14790-bib-0032] Manu‐Aduening, J.A. , Lamboll, R.I. , Ampong Mensah, G. *et al*. (2006). Development of superior cassava cultivars in Ghana by farmers and scientists: The process adopted, outcomes and contributions and changed roles of different stakeholders. Euphytica, 150, 47–61.

[ijfs14790-bib-0033] Mapiemfu‐Lamaré, D. , Ngome, F.A. , Eyenga, E.F. , Mbassi, J.E.G. & Suh, C. (2017). Harvesting date influences Cassava (Manihot Esculenta Crantz) yield and quality of based‐products. Current Research in Agricultural Sciences, 4, 75–83.

[ijfs14790-bib-0034] Mouafor, B.I. , Temegne, N.C. , Ngome, A.F. & Malaa, D. (2016). Farmer’s adoption of improved cassava varieties in the humid forest agro‐ecological zone of Cameroon. Greener Journal of Agricultural Sciences, 6, 276–284.

[ijfs14790-bib-0035] Ngueulieu, E.P. (2013). Incidences des migrations internes sur le développement local au Cameroun: cas de l’arrondissement de Banwa dans le Haut‐Nkam(Ouest‐Cameroun). Master Thesis, Faculty of Arts, Letters and Social Sciences, University of Yaoundé 1, Cameroon.

[ijfs14790-bib-0036] Njukwe, E. , Hanna, R. , Kirscht, H. & Araki, S. (2013). Farmers perception and criteria for cassava variety preference in Cameroon. African Study Monographs, 34, 221–234.

[ijfs14790-bib-0037] Njukwe, E. , Onadipe, O. , Thierno, D.A. *et al*. (2014). Cassava processing among small‐holder farmers in Cameroon: Opportunities and challenges. International Journal of Agricultural Policy and Research, 2, 113–124.

[ijfs14790-bib-0038] Nkuintchua, T. (2016). L’état des droits foncier des communautés en Afrique. Africa Community Rights Network. 100 p. http://www.cedcameroun.org/wp‐content/uploads/2016/12/letat‐des‐droits‐des‐communautes.pdf

[ijfs14790-bib-0039] Nweke, F. (2004). New challenges in the cassava transformation in Nigeria and Ghana. EPTD discussion papers 118. Washington, DC: International Food Policy Research Institute (IFPRI).

[ijfs14790-bib-0040] Ojulong, H. , Labuschangne, M.T. , Fregene, M. & Herselman, L. (2008). A cassava clonal evaluation trial based on a new cassava breeding scheme. Euphytica, 160, 119–129.

[ijfs14790-bib-0041] Olanrewaju, A.S. (2016). Effect of Garification (roasting) duration on the quality characteristics of Cassava *Gari* . Annals of Food Science and Technology, 17, 358–366.

[ijfs14790-bib-0042] Olanrewaju, A.S. & Idowu, O.E. (2017). Quality assessment of cassava *Gari* produced in some selected local governments of Ekiti State, Nigeria. American Journal of Food Science and Nutrition, 4, 36–41.

[ijfs14790-bib-0043] Olaoye, O.A. , Lawrence, I.G. , Cornelius, G.N. & Ihenetu, M.E. (2015). Evaluation of quality attributes of cassava product (gari) produced at varying length of fermentation. American Journal of Agricultural Science, 2, 1–7.

[ijfs14790-bib-0044] Olayide, P. , Large, A. , Stridh, L. *et al*. (2020). Gene expression and metabolite profiling of thirteen Nigerian cassava landraces to elucidate starch and carotenoid composition. Agronomy, 10, 424.

[ijfs14790-bib-0045] Oluwafemi, G.I. & Udeh, C.C. (2016). Effect of fermentation periods on the physicochemical and sensory properties of *Gari* . IOSR Journal of Environmental Science, Toxicology and Food Technology, 10, 37–42.

[ijfs14790-bib-0046] Onasoga, M.O. , Ayodele, D.O. & Oyeyipo, O.O. (2014). Chemical changes during the fortification of cassava meal (*Gari*) with African breadfruit (*Treculia africana*) Residue. Journal of Applied Sciences and Environmental Management, 18, 506–512.

[ijfs14790-bib-0047] Peprah, B.B. , Parkes, E. , Manu‐Aduening, J. , Kulakow, P. , van Biljon, A. & Labuschagne, M. (2020). Genetic variability, stability and heritability for quality and yield characteristics in provitamin A cassava varieties. Euphytica, 216, 31. 32055054 10.1007/s10681-020-2562-7 PMC6988135

[ijfs14790-bib-0048] Petsakos, A. , Prager, S.D. , Gonzalez, C.E. *et al*. (2019). Understanding the consequences of changes in the production frontiers for roots, tubers and bananas. Global Food Security, 20, 180–188.

[ijfs14790-bib-0049] Roszkowska, E. (2013). Rank ordering criteria weighting methods – a comparative overview. Optimum Studia Ekonomiczne 5, 14–33.

[ijfs14790-bib-0050] Sanginga, N. (2015). Root and Tuber Crops (Cassava, Yam, Potato andSweet Potato). An action Plan for Agricultural Transformation, Background Paper, feeding Africa, 21–23 Oct. 2015. 29pp.

[ijfs14790-bib-0051] Sanni, L.O. , Adebowale, A.A. , Awoyale, W. & Fetuga, G.O. (2009). Quality of *gari* (roasted cassava mash) in Lagos State. Nigeria. Nigerian Food Journal, 26, 125–134.

[ijfs14790-bib-0052] Sanoussi, A.F. , Yéyinou Loko, L. , Ahissou, H. *et al*. (2015). Diversity, physicochemical and technological characterization of elite cassava (Manihot esculenta Crantz) Cultivars of Bantè, a District of Central Benin. The Scientific World Journal, 2015, 674201. 26693522 10.1155/2015/674201 PMC4677020

[ijfs14790-bib-0053] Spencer, D.S.C. & Ezedinma, C. (2017). Cassava cultivation in sub‐Saharan Africa. In Achieving Sustainable Cultivation of Cassava Volume 1: Cultivation Techniques (edited by C. Hershey ) Cambridge, UK: Burleigh Dodds Science Publishing Limited.

[ijfs14790-bib-0054] Sureeyatanapas, P . (2016). Comparison of rank‐based weighting methods for multi‐criteria decision making. KKU Engineering Journal, 43(S3), 376–379.

[ijfs14790-bib-0055] Taiwo, K.A. & Fasoyiro, S.B. (2015). Women and cassava processing in Nigeria. International Journal of Development Research, 5, 3513–3517.

[ijfs14790-bib-0056] Teeken, B. , Olaosebikan, O. , Haleegoah, J. *et al*. (2018). Cassava trait preferences of men and women farmers in Nigeria: implications for breeding. Economic Botany, 72, 263–277. 30573920 10.1007/s12231-018-9421-7 PMC6267705

[ijfs14790-bib-0057] Thiele, G. , Dufour, D. , Vernier, P. *et al*. (2021). Review of varietal change in roots, tubers and bananas: consumer preferences and other drivers of adoption and implications for breeding. International Journal of Food Science and Technology, 56, 1076–1092. 10.1111/ijfs.14684 PMC7983933 33776222

[ijfs14790-bib-0058] Tofallis, C. (2014). Add or multiply? A tutorial on ranking and choosing with multiple criteria. INFORMS Transactions on Education, 14, 109–119.

[ijfs14790-bib-0059] Tohnain, N.L. & Bebnji, F. (2017). The use of technology by women gari producers in Bamunkumbit village, Northwest Region of Cameroon. IJRDO‐Journal of Agriculture and Research, 3, 37–52.

[ijfs14790-bib-0060] Tokula, M.H. & Ekwe, K.C. (2006). Utilization of improved cassava varieties among extension agents in Benue State, Nigeria. Journal Of Agriculture and Social Research (JASR), 6, 80–85.

[ijfs14790-bib-0061] Udofia, P.G. , Udoudo, P.J. , Eyen, P.J. & Udoekong, N.S. (2011). Optimizing *gari* quality attributes for different groups of consumers with response surface methodology. Journal of Agricultural Biotechnology and Sustainable Development, 3, 28–34.

[ijfs14790-bib-0062] Ukenye, E. , Ukpabi, U.J. , Chijoke, U. & Egesi, C. (2013). Physicochemical, nutritional and processing properties of promising new white and yellow fleshed cassava genotypes in Nigeria. Pakistan Journal of Nutrition, 12, 302–305.

[ijfs14790-bib-0063] Ukpabi, U.J. , Omodamiro, R.M. & Oti, E. (2012). Feasibility of using sealed polyethylene film in prolonged storage of *Gari* . Advances in Applied Science Research, 3, 1239–1243.

[ijfs14790-bib-0064] Ukwuru, M.U. & Egbonu, S.E. (2013). Recent development in cassava‐based products research. Academia Journal of Food Research, 1, 1–13.

[ijfs14790-bib-0065] Walker, T. , Alene, A. , Ndjeunga, J. *et al*. (2014). Measuring the effectiveness of crop improvement research in sub‐Saharan Africa from the perspectives of varietal output, adoption, and change: 20 Crops, 30 Countries, and 1150 Cultivars in Farmers’ Fields. Synthesis Report for Objectives 1 and 2 of Bill & Melinda Gates Foundation’s Diffusion and Impact of Improved Varieties in Africa (DIIVA) Project. Rome, Italy: CGIAR Independent Science and Partnership Council.

[ijfs14790-bib-0066] Wassmer, G. (2013). Evaluation des projets financés par le programme d’Amélioration de la Compétitivité des Exploitations Familiales Agropastorales (ACEFA): Cas de la région Sud du Cameroun. Master of Engineering thesis, 134 pp, ISTOM, Ecole Supérieure d’Agro‐Développement International, France.

[ijfs14790-bib-0067] Westby, A. (1991). Importance of fermentation in cassava processing. In: Proceedings of the Ninth Symposium of the International Society for Tropical Root Crops; 20–26 October 1991. Accra, Ghana.

[ijfs14790-bib-0026] Whitehead, A. , Vivian, J. , Lockwood, M. & Kasante, D. (2000). Gender and the expansion of non traditional agricultural exports in Uganda. Geneva, Switzerland: The United Nations Research Institute for Social Development (UNRISD). 63 p. https://www.unrisd.org/unrisd/website/document.nsf/(httpPublications)/832924CAD254DE9880256B67005B75CF?OpenDocument

[ijfs14790-bib-0068] World Bank, Food and Agriculture Organization and International Fund for Agricultural Development . (2009). Gender in Agriculture: Sourcebook. Washington DC: World Bank.

[ijfs14790-bib-0069] Wossen, A.T. , Girma Tessema, G. , Abdoulaye, T. *et al*. (2017). The Cassavamonitoring Survey in Nigeria: Final Report. Ibadan: IITA.

[ijfs14790-bib-0070] Xing, Y. , Hernández Nopsa, J.F. , Andersen, K.F. *et al*. (2020) Global cropland connectivity: A risk factor for invasion and saturation by emerging pathogens and pests. BioScience, 70, 744–758. 32973407 10.1093/biosci/biaa067 PMC7498352

[ijfs14790-bib-0071] Zundel, C. , Chibikom, R. , Scheidegger, U. , Nagel, P. & Hanna, R. (2010). Developing cassava cultivars based on farmers’ needs and on agro‐ecological conditions of North‐Western Cameroon. African Journal of Root and Tuber Crops, 8, 22–33.

